# Effectiveness of Self-Assembling Peptide (P11-4) in Dental Hard Tissue Conditions: A Comprehensive Review

**DOI:** 10.3390/polym14040792

**Published:** 2022-02-18

**Authors:** Ali Azhar Dawasaz, Rafi Ahmad Togoo, Zuliani Mahmood, Ahmad Azlina, Kannan Thirumulu Ponnuraj

**Affiliations:** 1 Department of Diagnostic Dental Sciences, College of Dentistry, King Khalid University, Abha 62529, Saudi Arabia; adwasaz@kku.edu.sa; 2 School of Dental Sciences, Universiti Sains Malaysia, Kubang Kerian 16150, Kelantan, Malaysia; zuliani@usm.my (Z.M.); azlinakb@usm.my (A.A.); 3 Department of Pediatric Dentistry and Orthodontic Sciences, College of Dentistry, King Khalid University, Abha 62529, Saudi Arabia; ratogo@kku.edu.sa; 4 Human Genome Centre, School of Medical Sciences, Universiti Sains Malaysia, Kubang Kerian 16150, Kelantan, Malaysia

**Keywords:** tooth remineralisation, dental enamel, dentin, P11-4 peptide, biomimetics, calcium hydroxyapatite

## Abstract

The limitations on the use of fluoride therapy in dental caries prevention has necessitated the development of newer preventive agents. This review focusses on the recent and significant studies on P11-4 peptide with an emphasis on different applications in dental hard tissue conditions. The self-assembling peptide P11-4 diffuses into the subsurface lesion assembles into aggregates throughout the lesion, supporting the nucleation of de novo hydroxyapatite nanocrystals, resulting in increased mineral density. P11-4 treated teeth shows more remarkable changes in the lesion area between the first and second weeks. The biomimetic remineralisation facilitated in conjunction with fluoride application is an effective and non-invasive treatment for early carious lesions. Despite, some studies have reported that the P11-4 group had the least amount of remineralised enamel microhardness and a significantly lower mean calcium/phosphate weight percentage ratio than the others. In addition, when compared to a low-viscosity resin, self-assembling peptides could neither inhibit nor mask the lesions significantly. Moreover, when it is combined with other agents, better results can be achieved, allowing more effective biomimetic remineralisation. Other applications discussed include treatment of dental erosion, tooth whitening and dentinal caries. However, the evidence on its true clinical potential in varied dental diseases still remains under-explored, which calls for future cohort studies on its in vivo efficacy.

## 1. Introduction

Tooth enamel is a complex structure comprising organic and inorganic components that produce the human body’s strongest mineralised structure. Hydroxyapatite crystals, which are calcium phosphate salts, make up the mineral composition of enamel [1], resulting in enamel prisms [2]. Dixit et al., in 2021 stated that dental caries results in focal disintegration of the mineralised tissues of teeth which is attributed to numerous cycles of demineralisation and remineralisation, with the intervening phases being either reversible or irreversible [3].

As early as 1982, Mizrahi stated that demineralisation is evident in early carious lesions known as white spot lesions (WSLs), and the presence of underlying porosities give them a milky appearance [4]. Demineralisation and remineralisation are natural processes that take place in the oral cavity. Boland, in 1999 found that during the caries process, there is increased removal of inorganic minerals from the periphery of the prisms due to its enhanced accessibility and solubility [5], which is followed by the dissolving of prism bodies [2]. Dental enamel is dissolved by the acids from meals, soft drinks, and bacteria found in plaque [6]. Madan et al. [7] in 2011 concluded that the saliva’s buffering effect aids in remineralisation by allowing calcium and phosphate ions to precipitate onto the tooth surface and produce new material. Another group of authors stated the most common early sign of dental caries progression is the appearance of WSLs or reversible regions of demineralisation [8].

Kidd, in 2004 stated that less invasive and more biological approaches such as remineralisation and regeneration of biological tissue have challenged the traditional treatment of cavitated lesions by drilling and filling. This merely involves replacing damaged tissue with foreign material, temporarily masking the process [9]. Hence, this raises the question of whether it is genuinely possible to reconstruct something which was made by nature and eventually destroyed during the caries process. Innes, as early as 2016, stated that there is emerging evidence and worldwide consensus in favour of utilising less invasive therapies that focus on lesion control rather than tissue removal [10]. Restorative treatment is limited to cavitated non-cleansable, carious lesions or restoring afflicted teeth function, form, and aesthetics [11].

When encountering demineralised WSLs, the primary goal must be to remineralise the lesions using a non-invasive method [12]. This necessitates the detection of these lesions in their early phases of development. Topical fluoride application has been popular for years to prevent enamel demineralisation and speed up the remineralisation process [13]. Gurunathan et al., in 2012 have reported that the utilisation of bioactive materials and calcium in the form of the Casein Phosphopeptide-Amorphous Calcium Phosphate (CPP-ACP) complex is one of the more recent innovations in the treatment of early carious lesions [14].

The self-assembling peptide was first discovered in 1989 due to curiosity-driven research similar to the chance discovery of X-ray and CRISPR for gene editing [15]. Since then, self-assembling peptides have been used in an array of applications ranging from surfactant materials to accelerated wound healing in regenerative medicine [15,16].

A self-assembling peptide molecule P11-4 has been studied extensively. It is a rationally designed synthetic peptide of 11 amino acids that undergoes hierarchical self-assembly into B-sheet tapes, ribbons, fibrils and fibres [17]. It exists as unimers of random coil conformations in water above pH 7.5 but at low pH adopts an antiparallel β-sheet conformation. It also self-assembles under physiological conditions in a concentration-dependent manner [18]. It has been demonstrated that incorporation of Glu- or Orn- into the primary structure could enable rapid and reversible self-assembly by simply changing the pH [17]. Li and colleagues studied the role of self-assembling peptides in enamel remineralisation based on a biomimetic approach [19], whose main goal is to replicate the natural process of enamel mineralisation. It was also reported in 2016 that non-collagenous proteins with a negative charge play a function in attracting positively charged calcium ions during the natural process of mineralisation [20] and that the negative charge acts as a nucleation site, and mineral crystal formation is accomplished through the growth and fusion of mineral nuclei. In addition to the use of P11-4 peptide on enamel, its applicability on dentin tissues has lately been investigated. De Sousa et al., in 2019 assessed P11-4’s interaction with organic dentin components, as well as its impact on proteolytic activity, mechanical aspects of the bonding interface, and nanoleakage evaluation in simulated caries-affected dentin. P11-4 interacts with collagen type I fibres, improving the integrity of the hybrid layer generated by artificial caries-affected dentin and enhancing collagen fibre resistance to proteolysis [21].

This review describes the technology of self-assembling peptides and the mechanism of 3D scaffold construction, emphasising the notion of de novo hydroxyapatite crystal formation in response to the administration of peptide P11-4, assisting in enamel/dentin remineralisation after the initiation of dental caries. In addition, its applicability in dentinal hypersensitivity and dental erosion is discussed. Bearing that in mind, the following review question was addressed ‘What are the different applications of P11-4 self-assembling peptide in dental hard tissue (Enamel/Dentin) conditions’?

For searching all relevant studies on the efficacy of P11-4 self-assembling peptide in various dental hard tissue conditions, PubMed, Scopus, Embase, Web of Science databases was searched as follows: (((“P11-4 peptide” [Supplementary Concept]) AND “Dental Enamel” [Mesh]) OR “Dentin Sensitivity” [Mesh]) OR “Tooth Erosion” [Mesh].

The literature search was carried out in the Journal of Dental Research, Journal of Endodontics, and Journal of Conservative Dentistry to ensure a complete screening process. Review articles were used as additional sources of references for further information. All included studies were checked for the availability of full text. The search was mainly focused on mapping existing literature. The search span had articles until January 2022 published in English. A total of 7845 full-text articles were found. Duplicate records were removed using Mendeley (Elsevier Inc., USA), and 868 free full-text articles were retrieved. Based on our inclusion criteria, 35 articles were included for review. The details of the included articles are presented in Table 1. Most of the articles focused on evaluating P11-4 peptides on remineralisation of enamel caries [22,23,24,25,26,27,28,29,30,31,32,33,34,35,36,37,38,39,40,41,42,43,44,45,46,47,48,49]. Only three articles were found to have studied its effects on dentin [21,50,51], while two others assessed remineralisation in dental erosion [52,53], and one assessed its efficacy in tooth whitening [54,55].

## 2. P11-4 Self-Assembling Peptide

Acar et al., in 2017 described the roles of proteins and peptides in the human body, which can fold into various shapes, making them valuable biomaterials. The building blocks of peptides are amino acids [56]. They also suggested that amino acid side chains with a terminal -COOH or -NH_2_ can be placed [16]. Aggeli et al., in 2003 stated that there is a controlled interaction between adjacent peptides [17], and this interaction can self-organise into different structures [56]. In 2017, Fan et al. referred to this as molecular self-assembly. In addition, they stated that specific peptides, such as the α-helical peptide, β-sheet peptide, amphiphilic peptide, cyclic peptide, and dipeptide, can self-assemble [57]. The lower occurrence of side effects and stable drug release are also advantages of peptide-based self-assembled structures [58,59]. Self-assembly of these structures can result in the formation of nanostructures, including nanofibers, nanotubes, and nanovesicles [60]. In 2007, Gelain et al. itemised the use of these self-assembled nanostructures as scaffolds in the field of regenerative medicine, 3-D tissue cell culture and drug delivery systems were reported [61]. The self-assembling peptide P11-4 is one of the promising biomimetic alternatives for enamel remineralisation. P11 peptide group performs one-dimensional, hierarchical self-assembly when the concentration of peptide monomer approaches the critical monomer concentration (C*). Within seconds, micrometre-long- β sheet nanotapes and ribbons are formed and then assembled to create fibrils and edge-to-edge fibres during the next 24 h [34,62].

The glutamine residues at the end of the chain increase hydrophobic interactions and hydrogen bonding. The presence of arginine in the chemical structure results in a positive net charge, allowing for antiparallel β-sheet formation. Since glutamate residues are negatively charged at higher pH in aqueous solution, electrostatic repulsion prevents efficient β -sheet production [18]. However, it was discovered that sodium ions could buffer the repulsive effects of negatively charged residues at physiological conditions (pH 7.4, NaCl 130 mM). P11-4 undergoes self-assembly as a result of the ionic interaction between the negatively charged glutamic acid and positively charged arginine [63].

P11-4 features four negatively charged Glu-residues that could act as Ca^2+^ binding sites when assembled into fibres. The distance between these sites is 9.4, which is close to the position of columnar Ca^2+^ ions in the HAP crystal lattice [24]. Furthermore, Firth et al. used energy-dispersive X-ray examination and revealed that the crystals’ Ca/P molar ratios are consistent with HAP-1.67 [64]. Therefore, this anionic peptide can be used as a low viscosity, injectable monomer solution that can infiltrate demineralised enamel and gel quickly at pH levels below 8.0 [64]. Furthermore, the freshly constructed three-dimensional scaffold has a strong chemical interaction with the tooth surface. Therefore, it could act as a template for HAP nucleation and deposition within the lesion, mimicking the role of enamel matrix proteins [65].

## 3. The Rationale behind the Use of P11-4

In the vast majority of instances, untreated or inadequately treated incipient caries results in the formation of a cavity in the tooth, which must be treated invasively. A filling might last anywhere from 10 to 15 years. Following that, a more extensive filling is routinely placed, which frequently results in tooth loss. It has been reported [35,66] that fluoride is the main reason for the reduction in caries because of its cariostatic potential. Despite its effectiveness in slowing the advancement of caries, it has several drawbacks. Fluoride does not entirely eliminate caries. Moreover, in-depth mineralisation does not occur [35]. This necessitates the use of therapy to regenerate a mineral deficiency in the tooth enamel due to a carious lesion [21,23,24,26,32,38,40,67,68] and could be termed as ‘Guided Enamel Remineralisation’ (GER). Dentin, too, has the potential to undergo such biomimetic remineralisation [21,50,51].

## 4. Method of Application of P11-4 Peptide

It is pertinent to remove the superficial pellicle using 2% sodium hypochlorite followed by the application of 35% phosphoric acid for 20 s. After cleaning and drying the teeth, the surface must be assessed for the presence of open pores. This is intended for allowing the material to penetrate the lesion and initiate the process. Fluorides and other remineralisation-promoting chemicals can also aid in this process, as observed in previous literature [25,46,69]. Fluoridation with products containing more than 5000 ppm, on the other hand, should not be carried out immediately after application. As the process of remineralisation is time-dependent, it is necessary to administer the peptide numerous times over the course of 3–6 months to achieve the beneficial effect. However, a study by Brunton found that a single application is associated with significant enamel regeneration, presumably by promoting mineral deposition within the subsurface tissue [34].

## 5. Mode of Action

Aggeli et al. described the mode of action of the P11-4 peptide. Its chemical structure (Ace-Gln-Gln-Arg-Phe-Glu-Trp- Glu-Phe-Glu-Gln-Gln-NH_2_) is made up of five amino acids: arginine, tryptophan, phenylalanine, glutamine, and glutamic acid [70]. It is also called oligopeptide 104. They also described that the 11 amino acid peptide P11-2 is modified to allow the self-assembly of peptides in response to pH changes. The glutamine (Gln) residues of peptide P11-2 are organised in a specific order. These residues have side chains that interact and cause β-sheet formations to form [71]. By replacing the glutamic acid residues at positions 5 and 7 with glutamine residues, peptide P11-4 was induced to remain monomeric at high pH and transform to a nematic gel at low pH [70].

As early as 2006, Davies et al. described the formation of peptide fibres from the β-sheet structure that self-assembles to generate nanotapes, the simplest kind of hierarchical structure. These tapes have a helical structure due to their twisting and bending. Nanoribbons are formed when nanotapes are intertwined. The nanoribbons do not have the same helical structure as nanotapes but instead have a saddle curvature. This occurs because the tapes’ bending, and twisting must be reduced to promote stacking [62]. Nanoribbon stacking results in the production of nanofibrils. The number of ribbons that can be stacked is determined by the balance between the untwisting of nanoribbons involved in stacking and the increase in the attraction energy of these ribbons [57]. A ribbon with a short twist angle will have a large pitch. If the magnitude of attraction energy is large enough, the ribbon will untwist completely, causing stacking and the production of a two-dimensional crystal [60,62]. A ribbon with significant twist angles and a smaller magnitude of attraction energy will not produce fibrils; instead, the equilibrium structure in the solution will be a ribbon. Low to moderate twisting angles and low to intermediate attraction energy are thus required to produce separate fibrils. Edge-to-edge entwining is possible with stable fibrils. Peptide fibres are thus formed [62], serving as a scaffold for the de novo creation of hydroxyapatite crystals [34].

**Figure 1:**
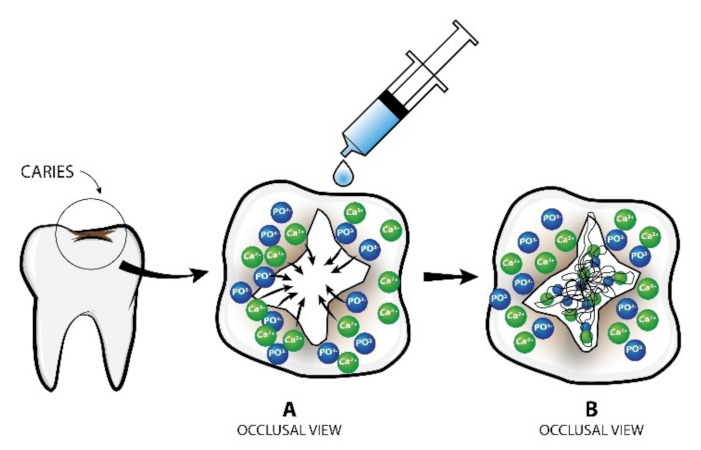
Mode of action of P11-4 on dental caries. (**A**) Calcium hydroxyapatite crystals around the lesion are attracted inwards and interact with peptide molecules; (**B**) Formation of new hydroxyapatite crystals within the scaffold created.

The mechanism of action of the P11-4 peptide (Figure 1) shows the formation of a hierarchical self-assembly unit. Kirkham et al., in 2007 described its conversion to nanostructures, thereby constructing a scaffold under appropriate environmental circumstances [25]. The presence of cations and a low pH of 7.4 characterise a carious lesion. Peptide P11-4 self-assembles under these conditions [34]. By undergoing self-assembly in one dimension, it produces a β-sheet structure. Intermolecular hydrogen bonding and interactions between side chains are involved in this self-assembly process [25]. Fan et al., in 2017, described the β-sheet structure, which is amphiphilic because it has a peptide sequence with alternating hydrophobic and hydrophilic amino acids. The amphiphilic feature of the β-sheet structure drives its self-assembling property [57].

It was in 2006 that the interaction between peptide aggregates leading to the formation of either flexible 3D structure or more rigid anisotropic gel was described [62]. As the peptide concentration grows, so does the number of aggregates generated and the average length of the peptides. At a given concentration (Cgel), the interaction between the aggregates can be seen. When the concentration of a solution exceeds that of Cgel, it is considered a semi-dilute state. The more flexible tapes and ribbons create a sponge-like 3D structure, which leads to gelation. On the other hand, the more rigid fibrils align into nematic domains and join to form an anisotropic gel structure. This method shifts the liquid from isotropic to the nematic condition [62].

Previous researchers have demonstrated that peptide P11-4 has a low viscosity and is an isotropic liquid at high pH [62]. It changes to a nematic gel state during the self-assembly process, occurring at a pH range of 6.8–7.2 [17]. They used rheological measures to investigate the transition of nematic to isotropic fluids. According to their findings, the self-assembling peptide P11-4 remains monomeric and shows Newtonian behaviour at higher pH values. It showed an intermittent behaviour between isotropic liquid and nematic gel in the pH range of 6.9 to 7.3, which is known as the biphasic region. The viscosity falls at pH 6.6, and it transforms into a nematic state with viscoelastic fluid features; at pH 2, it transforms into a nematic gel with low yield stress. Between pH 2.0 and pH 13.0, the self-assembling peptide exists in four distinct states [17]. They could also create an instantaneous changeover between the nematic gel phase and isotropic fluid phase by appropriately introducing an acid or a base. However, after four ‘pH jumps’, the gel flocculated due to increased ionic strength [48].

Wierichs et al., in 2017, discussed the disadvantages of the self-assembling peptide technique. They concluded that the nematic form of a self-assembling peptide undergoes flocculation in oral environmental settings, where the pH fluctuates due to alternate demineralising and remineralising cycles. This flocculated condition of the self-assembling peptide is relatively inert and may obstruct the remineralisation process [39]. Furthermore, they also stated that during the remineralisation process, incorporating these flocculates into the enamel impacts the diffusion of calcium, phosphate, and fluoride ions to the enamel surface. As a result, during later phases of demineralisation, the availability of fluoride ions is reduced [39].

## 6. Evaluation of Efficacy of P11-4 Peptide

In vivo clinical evaluation of successful treatment by P11-4 peptide is assessed by multiple methods. Welk et al. assessed using impedance measurement by CarieScan Pro and morphometric measurement (in mm^2^) of the lesion [38], tactile sensation using dental mirror and explorer. DIAGNOdent has also been used by some authors [27]. International Caries Detection and Assessment System (ICDAS) II was used to assess qualitative improvement in remineralisation [27].

**Figure 2:**
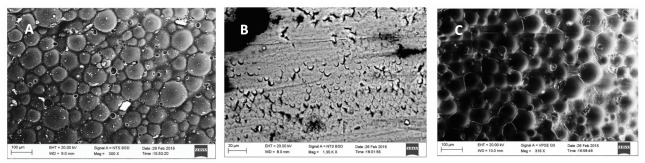
Scanning electron microscopy images (**A**) Normal enamel surface; (**B**) Demineralised enamel; (**C**) P11-4 treated enamel surface after demineralisation [35] (Reproduced with permission).

In vitro studies on P11-4 peptide were assessed using Scanning Electron Microscopy (SEM), Atomic Force Microscopy (AFM) with microindentation [72], DIAGNOdent, micro-CT [37,43,73], infrared (IR) spectroscopy, circular dichroism (CD) [74]. AFM based nanoindentation is a valuable tool to investigate the demineralisation and remineralisation of surface softened enamel with high accuracy [72]. Soares et al., 2017 described enamel remineralisation by P11-4 peptide using SEM as shown in Figure 2 [35].

Structural similarity between sound enamel and P11-4 treated enamel suggested biomimetic remineralisation by nucleating HA crystals. Soares et al. have also reported greater surface mean hardness values compared to Casein Phosphopeptide-Amorphous Calcium Phosphate Fluoride (CPP ACPF), Bioactive Glass (BAG) and fluoride enhanced hydroxyapatite (HA) gel [35]. Similar results were reported by Jablonski et al., in 2014 [44].

## 7. Uses of P11-4 Peptide in Dental Hard Tissue Conditions

### 7.1. Early Enamel Caries Remineralisation

Brunton et al., in 2013 [34] found porosities in a WSL of the enamel and reported that due to its low viscosity, monomeric peptide P11-4 penetrates these porosities when applied. The peptide self-assembles into a viscous fibrous scaffold under the influence of the circumstances seen in a carious environment. The anionic groups in peptide P11-4 attract calcium ions and can precipitate hydroxyapatite crystals from scratch. The nucleator pulls ions out of tissue fluids and organises them into a crystalline structure. The crystals will only grow once the crucial nuclei have been stabilised. The scaffold matrix is responsible for this stabilisation. This mechanism is similar to what happens naturally prior to tooth eruption when the enamel matrix proteins self-assemble to guide hydroxyapatite crystal precipitation [34].

Ex vivo research and clinical trials on class V early carious lesions have looked into the effect of self-assembling peptides. Brunton et al. also stated that early enamel smooth surface lesions have been shown to benefit from the use of self-assembling peptide P11-4 (Curodont^®^). The peptide’s main effects are seen within the first thirty days of treatment and are manifested as a reduction in the size of the lesion. However, the pitfall of this study is the absence of a control group [34]. After the peptide builds a new enamel matrix, calcium and phosphate ions from saliva are incorporated to remineralise the teeth [44]. This effect is maintained by using the remineralising paste regularly. Schlee et al., in 2018, studied the use of P11-4 peptide on proximal caries. They stated that once a proximal carious lesion reaches the dentin, the risk of cavitation increases dramatically. A restorative procedure for such a lesion would result in the tooth’s integrity being compromised. As a result, it’s critical for a clinician to try to prevent a proximal carious lesion from progressing into dentin. The use of self-assembling peptide P11-4 has resulted in the regression of initial proximal carious lesions, delaying or avoiding the necessity for restorative procedures [30].

A contrasting study by Golland et al., in 2017 on bovine teeth concluded that application of P11-4 peptide on demineralised bovine enamel did not lead to increased fluorescence measured using quantitative laser fluorescence, indicating either lack of remineralisation or irregular crystals [42]. Additionally, Memarpour et al., (2021) revealed P11-4 peptide treated primary teeth had the lowest percentage of surface enamel microhardness. In addition, the mean calcium/phosphate weight percentage ratio of P11-4 was significantly lower than the others (*p* < 0.001) [29]. Recently, a study by Wahba and colleagues reported on the inability of P11-4 peptide to remineralise caries in deciduous teeth [75]. However, some studies have concluded the beneficial effects of P11-4 peptide combined with fluoride varnish over the use of fluoride varnish alone [32,45]. Furthermore, another study reported that P11-4 peptide worked better when it was combined with either fluoride or CCP-ACPF than when used alone [46].

### 7.2. Orthodontic Treatment-Induced Caries

Demineralisation can occur near brackets during fixed orthodontic treatment. Early enamel alterations may progress, and WSLs may arise if appropriate preventative strategies are not used. According to a meta-analysis, 45.8% of patients developed new carious lesions while undergoing orthodontic treatment [76]. High treatment demand and the prevalence of biofilm-related problems, according to some experts [77], make orthodontic treatment a possible public health issue.

Although fluoride has been found to successfully prevent tooth cavities [78,79], when the caries lesion has developed to the clinically evident WSL, and the best probable outcome is an arrest of the lesion’s activity, there are restrictions to using fluoride [66]. In 2020, Jablonski et al. studied the use of the P11-4 peptide in patients with a high caries risk, such as those receiving orthodontic treatment. They came to the conclusion that the mineral gain effectiveness of fluoride varnish is superior to the one-time application of fluoride varnish. The benefit is that it improves enamel remineralisation, which is especially important in these patients because fluoride alone may not be enough [45]. Another study by Knaup et al., in 2021 concluded that the shear bond strength was not affected by using the caries-protecting P114 peptide before attaching brackets. As a result, preparation of the enamel surface with P114 peptide prior to bracket insertion is a viable option [26].

### 7.3. Dentinal Caries

A perusal of literature showed only a study by de Sousa et al. [21] that analysed the applicability of the self-assembling peptide P11-4 in the dentin tissue with organic components of the dentin matrix. The results demonstrated a new and promising ability of the self-assembling peptide P11-4 of binding type I collagen. Such characteristics increased the fibrils’ width and ultimately increased the immediate resistance of collagen type I fibres against collagenase activity. This could probably be due to P11-4 properties as a collagen binder, leading to the reinforcement of the bonding interface and the inhibition of collagen proteolysis at the hybrid layer. Atomic force microscopy of dentin samples showed dry P11-4 hydrogels with parallel nanofibrillar structures. The mean collagen type I fibre widths in the presence of P11-4 peptide increased from 30 to 330 nm [21].

### 7.4. Hypersensitivity of The Dentin

Two studies were found on hypersensitivity treatment using self-assembling peptides [50,51]. Dentine hypersensitivity (DH) is widely believed to occur as a result of fluid flow within exposed dentinal tubules in the tooth surface. Most treatments are designed to occlude these tubules. In order to determine the efficacy of self-assembling peptide P11-4 in treating dentinal hypersensitivity, many randomised clinical trials have been conducted. Due to hydroxyapatite binding sites, the self-assembling peptide P11-4 has a high affinity for the dentinal surface. As a result, electrostatic interactions bind the matrix to the tooth, due to which the dentinal tubules are occluded, and dentinal hypersensitivity is reduced due to these interactions [51]. Another in vitro study investigated the ability of a novel self-assembling peptide matrix gel with calcium phosphate in effectively occluding dentine tubules compared to selected desensitising toothpastes [50]. The ability of the desensitising gel and toothpastes to occlude the dentine tubules was assessed and compared before and after brushing using Scanning Electron Microscopy (SEM) on both etched and fractured dentine surfaces. The self-assembling peptide matrix gel demonstrated a more significant reduction in the number of open tubules compared to the other desensitising toothpastes. Reductions in the hydraulic conductance measurements were observed to be 55.1 (± 12.5%) [50].

### 7.5. Erosion of The Enamel

Early reference of causative factor for enamel erosion dates back to 1975 by Geddes, who suggested increased consumption of highly acidic soft drinks and fruit juices [80]. Under scanning electron microscopy, these erosions can be seen as surface roughness and abnormalities. The enamel is protected using the self-assembling peptide P11-4 before or after exposure to such acidic environments [52]. It slows the deterioration of enamel and helps with remineralisation [53]. However, Attin et al. reported that there was no anti-erosive effect and as well as no significant difference from the untreated control group [81].

## 8. Perspective and Conclusions

Recent examples of many successful clinical trials of self-assembling peptide P11-4 in initiating regenerative capacity for dental hard tissues have provided a glimpse of its widespread applications in future. As this peptide holds potential for a breakthrough in dentistry in guided enamel remineralisation, further research is to be directed towards the effects of self-assembling peptides on the dentinal structures of the tooth.

It is germane to remember that remineralisation in vitro can be substantially different from changes in the oral cavity in vivo. As a result, direct extrapolations to clinical settings must be carried out with caution. Hence, future in vivo cohort studies on treated surfaces may be undertaken to unfold the long-term effects of oral environment on the clinical longevity of treated tooth surfaces. It also underscores further exploration of P11-4 peptide in regenerative therapy of human periodontal tissue defects. The authors also suggest the use of this technology in enhancing the bond strength prior to orthodontic treatment.

The promising effect of P11-4 on early enamel caries by guided enamel remineralisation is evidenced from previous studies. Some reports have doubted its beneficial effects when used alone; however, it is also pertinent to note that some studies have reported better outcomes when combined with other agents than when used alone. This leads to the inference that the evidence to draw a concrete conclusion on its true clinical potential still remains under-explored.

## Figures and Tables

**Table 1:** Functions of Self-assembling Peptide P11-4.

S. No.	Study Conducted	Study Outcome	Type of Dental Tissue	Reference
1	P11-4 with fluoride varnish on permanent molars	Clinically improvement at 3 and 6 months. The laser fluorescence readings and visual analogue scale scores were significantly lower and showed regression of caries	Enamel	[22]
2	P11-4 with fluoride varnish followed up for 360 days	The carious lesion mean size (SD) in the test group relative to baseline decreased to 0.936 (0.127) at 30 days and 0.862 (0.352) at 360 days	Enamel	[23]
3	Clinical efficacy of a single application of P11-4 on early enamel lesions followed up for 180 days	Treatment with P11-4 significantly decreased lesion size after 30 days	Enamel	[34]
4	In vitro study on enamel erosion produced by a soft drink using Atomic Force Microscopy and Scanning Electron Microscopy	Significant differences were recorded when comparing softened enamel with softened enamel further remineralised with biomimetic self-assembling peptides and enamel treated with it between two acid attacks	Enamel	[52]
5	In vitro interaction of P11-4 with the organic dentin components and its effect on the proteolytic activity, mechanical properties of the bonding interface, and nanoleakage evaluation to artificial caries-affected dentin	P11-4 binds to collagen type I fibres, increasing their width from 214 ± 4 nm to 308 ± 5 nm. It also increased their resistance against the proteolytic activity of collagenases. There was enhanced micro tensile bonding strength of the bonding interface, reaching values close to sound dentin; however, it decreased after six months of water storage	Dentin	[21]
6	Quantification of artificial caries sub-surface enamel lesion remineralisation on human molar using microCT in vitro	The mean mineralisation coefficient reached 35.5% for the peptide-treated specimen compared to 11.5% for the control	Enamel	[40]
7	In vivo effectiveness of monomeric self-assembling peptide P11-4 (SAP P11-4) in combination with fluoride varnish or polymeric self-assembling peptide matrix (SAPM) in treatment of non-cavitated occlusal caries	Laser fluorescence changes demonstrated superior results for P11-4 with fluoride and SAP P11-4 with SAPM as compared to control (only fluoride). ICDAS-II codes and Nyvad Caries Activity at day 360 also showed superior caries inactivation	Enamel	[41]
8	Remineralisation potential of a single application of P11-4 or acidic fluoride solution using quantitative light-induced fluorescence (QLF) in vitro on bovine enamel	Application of self-assembling peptides on demineralised bovine enamel did not lead to increased fluorescence using QLF, indicating either lack of remineralisation or irregular crystals	Enamel	[42]
9	The ability of a P11-4 with calcium phosphate in effectively occluding dentine tubules compared to other desensitising toothpastes in vitro	P11-4 demonstrated a more significant reduction in the number of open tubules compared to the other desensitising toothpastes	Dentin	[50]
10	Adhesion and whitening effects of a combination of P11-4 self-assembling peptide and hydroxyapatite (peptide-HAP) on bovine enamel	The peptide-HAP suspension is a mild tooth whitener, and the adhesion of peptide-HAP to enamel is concentration-dependent	Enamel	[54]
11	Regeneration of demineralised tooth tissue on smooth surfaces using DIAGNOdent (DD) and VistaProof (VP) fluorescence systems and SEM in vitro	P11-4 proved to be efficacious, and SEM images revealed large areas of remineralised enamel surface in 93% of the samples	Enamel	[44]
12	In situ ability of P11-4 (SAPM) to remineralise artificial initial caries lesions compared to fluoride varnish on bovine enamel exposed to the human oral environment	P11-4 (containing fluoride) demonstrated superior remineralisation potential compared to fluoride varnish alone and has added potential in high caries risk patients	Enamel	[43]
13	In vitro efficacy of the P11-4 for remineralisation combined with fluorides, compared to the application of fluoride varnish alone	Application of P11-4 with fluoride varnish was superior to the use of fluorides alone for remineralisation of enamel adjacent to brackets	Enamel	[45]
14	In vitro efficacy of P11-4 on enamel remineralisation combined with CPP-ACPF or fluoride using Surface microhardness (SMH) and scanning electron microscope (SEM)	The complementary effect was obtained after combining self-assembling peptide with CPP-ACPF or fluoride showing the highest remineralising potential even after four weeks	Enamel	[46]
15	In vitro assessment of the mechanism of action of P11-4 peptide in remineralising enamel	P11-4 can facilitate the subsurface regeneration of the enamel lesion by supporting de novo mineralisation in a similar mode of action as has been shown for the natural formation of dental enamel	Enamel	[24]
16	Effect of an anionic peptide applied to caries-like lesions in human dental enamel under simulated intra-oral conditions of pH cycling	Peptide treatment significantly increased net mineral gain by the lesions due to both increased remineralisation and inhibition of demineralisation over a five-day period. It was also capable of inducing hydroxyapatite nucleation de novo	Enamel	[25]
17	In vitro study to investigate P11-4 (SAP P11-4) on the shear bond strength of metal brackets	The application of the caries protective SAP P11-4 before bonding of brackets did not affect the shear bond strength. Hence can be considered before bracket insertion	Enamel	[26]
18	In vivo effectiveness of the SAP11-4 or tricalcium phosphate fluoride (TCPF) in remineralisation of WSLs in young permanent teeth	SAP11-4 treatment showed superiority in remineralisation of enamel compared to TCPF	Enamel	[27]
19	Evaluated fluoride varnish, enamel matrix protein, and self-assembling peptide derivatives with varying chemical compositions on remineralisation of white spot lesions in vitro using quantitative light-induced fluorescence (QLF) on bovine enamel	P11-4 and Clinpro XT were effective in diminishing the fluorescence loss and lesion area compared to the Duraphat, Enamel Pro fluoride varnishes, and Emdogain at different time points	Enamel	[28]
20	In vitro remineralisation of enamel caries lesions using the self-assembling peptide P11-4 associated with fluoride, CPP-ACP and bioactive glass	The mean calcium/phosphate weight percentage ratio of P11-4 was significantly lower than the others	Enamel	[29]
21	Clinical study to investigate SAPM gel compared to 8% arginine and calcium carbonate toothpaste for treatment of dentin hypersensitivity	Significant reduction of dentin hypersensitivity, with the patient questionnaire indicating higher patient satisfaction	Dentin	[51]
22	Effect of P11-4 on non-cavitated initial proximal carious lesions 12 months after treatment	Radiographic and digital subtraction analyses suggest that initial proximal carious lesions can regress after treatment with P11-4	Enamel	[30]
23	Re-hardening potential of enamel matrix derivatives (EMD) and self-assembling peptides in vitro	A significant re-hardening up to 125 pm was observed	Enamel	[31]
24	In vivo efficacy of self-assembling peptide P11-4 compared to placebo or fluoride varnish	P11-4 treatment resulted in superior caries regression compared to either placebo or fluoride varnish	Enamel	[32]
25	In vitro regeneration of enamel in natural early caries lesions and evaluated over 50 days by photothermal radiometry and luminescence	P11-4 promoted the regeneration of early caries	Enamel	[33]
26	Effect of P11-4 to remineralise artificial carious lesions in enamel in vitro using a 30-day pH cycling model through surface microhardness analysis and SEM	P11-4 remineralised the enamel lesions more effectively, and SEM photomicrographs demonstrated either amorphous crystals or particles scattered on the surface or lines of remineralisation along the prismatic borders	Enamel	[35]
27	Ultrasonography to evaluate the effect of the self-assembling peptide P11-4 on acid erosion prevention on bovine enamel	P11-4 peptides on erosive lesions can improve remineralisation	Enamel	[53]
28	Remineralising efficacy of NovaMin, CPP-ACP, silver diamine fluoride (SDF), and P11-4	Self-assembling peptides showed maximum remineralisation in tested specimens followed by CPP-ACP, SDF, and NovaMin	Enamel	[36]
29	Compared P11-4 with casein phoshopeptide-amorphous calcium phosphate fluoride (CPP-ACFP) and sodium fluoride (NaF) on artificial caries lesions using DIAGNOdent and micro-computed tomography (microCT) in vitro	P11-4 showed the best remineralisation efficacy, followed by CPP-ACFP and NaF	Enamel	[37]
30	Evaluated the effect of P11-4 (SAP) in the therapy of initial smooth surface caries (white spot lesions, WSL) following orthodontic multibracket treatment	Superior remineralisation of the subsurface lesions with P11-4 peptide compared with the control teeth.	Enamel	[38]
31	Compared novel treatment methods regarding their ability to hamper demineralisation in artificial enamel caries using pH-cycling model	self-assembling peptides could neither inhibit lesion progression nor mask the lesions considerably as compared to other treatment methods	Enamel	[39]
32	Effect of self-assembling peptide—Curodont on microhardness of bleached enamel surface	P11-4 exhibited a significant difference in the remineralising bleached enamel surface	Enamel	[55]
33	P11-4 after orthodontic treatment	Self-assembling peptide proved to be effective in WSLs after orthodontic treatment	Enamel	[47]
34	P11-4 in acute and chronic incipient caries lesions	The use of self-assembling peptides on white spot and brown spot caries lesions increased the hardness of these lesions in the deeper layers up to 275 µm	Enamel	[49]
35	Comparative evaluation of resin infiltration with P11-4 peptide for non cavitated smooth surface lesions	Moderate lesion regression was observed with P11-4 peptide	Enamel	[48]

## Data Availability

Not applicable.
